# Hypofractionated palliative radiotherapy for advanced ethmoid sinus squamous cell carcinoma: a case report

**DOI:** 10.1093/bjrcr/uaag037

**Published:** 2026-08-22

**Authors:** Osamu Tanaka, Naoki Isobe, Takuya Taniguchi, Takuji Kiryu, Chihiro Ikemoto, Chiyoko Makita, Masayuki Matsuo

**Affiliations:** Department of Radiation Oncology, Asahi University Hospital, Gifu, 501-1151, Japan; Department of Home Medical Care, Medical Corporation Kagayaki Comprehensive Home Medical Care Clinic, Gifu, 501-6014, Japan; Department of Radiation Oncology, Asahi University Hospital, Gifu, 501-1151, Japan; Department of Radiology, Asahi University Hospital, Gifu, 500-8523, Japan; Department of Radiation Oncology, Asahi University Hospital, Gifu, 501-1151, Japan; Department of Radiation Oncology, Gifu University Hospital, Gifu, 501-1194, Japan; Department of Radiation Oncology, Gifu University Hospital, Gifu, 501-1194, Japan

**Keywords:** ethmoid sinus carcinoma, hypofractionated radiotherapy, palliative care, squamous cell carcinoma, case report

## Abstract

Ethmoid sinus cancer is a rare malignancy, that is, often diagnosed at an advanced stage, making surgical intervention challenging. Herein, we report a case of advanced ethmoid sinus squamous cell carcinoma in a female patient in her 90s. The patient presented with right-sided proptosis, ptosis, diplopia, and nasal obstruction. She was considered unfit for radical surgery or chemoradiotherapy because of her age and other comorbidities. She underwent hypofractionated palliative radiotherapy (35 Gy in 5 fractions) targeting the clinical tumor volume only. Clinically meaningful symptom relief and substantial tumor shrinkage were observed post-treatment, with minimal acute toxicities, although conclusions regarding treatment efficacy should be interpreted cautiously given the single-patient nature of this report. This case illustrates the potential of hypofractionated radiotherapy as a palliative option for patients deemed unsuitable for radical treatments.

## Introduction

Among paranasal sinus tumors, ethmoid sinus cancer is a relatively rare malignancy, with an estimated annual incidence of approximately 70-100 cases in Japan. It is often locally advanced at diagnosis, with invasion into the orbit or anterior cranial base, making surgical resection difficult.^1–3^ Although the standard treatment for this cancer type involves a multidisciplinary approach combining surgery and radiotherapy, radical interventions may be unfeasible for older patients or those with poor general health.

In such cases, hypofractionated high-dose radiotherapy over a short period for local control and symptom relief has gained attention as a practical strategy to maintain patients’ quality of life (QOL).^4–7^ Herein, we report a case of advanced ethmoid sinus squamous cell carcinoma (SCC) that was deemed inoperable and treated with palliative hypofractionated radiotherapy at 35 Gy in 5 fractions, which resulted in tumor shrinkage and symptomatic improvement. To the best of our knowledge, this represents one of the few documented cases of hypofractionated palliative radiotherapy specifically for ethmoid sinus SCC in a very elderly patient (age >90 years) with multiple comorbidities, demonstrating the potential feasibility and clinical value of this approach when conventional treatment is unfeasible.

## Clinical presentation

**Figure 1:**
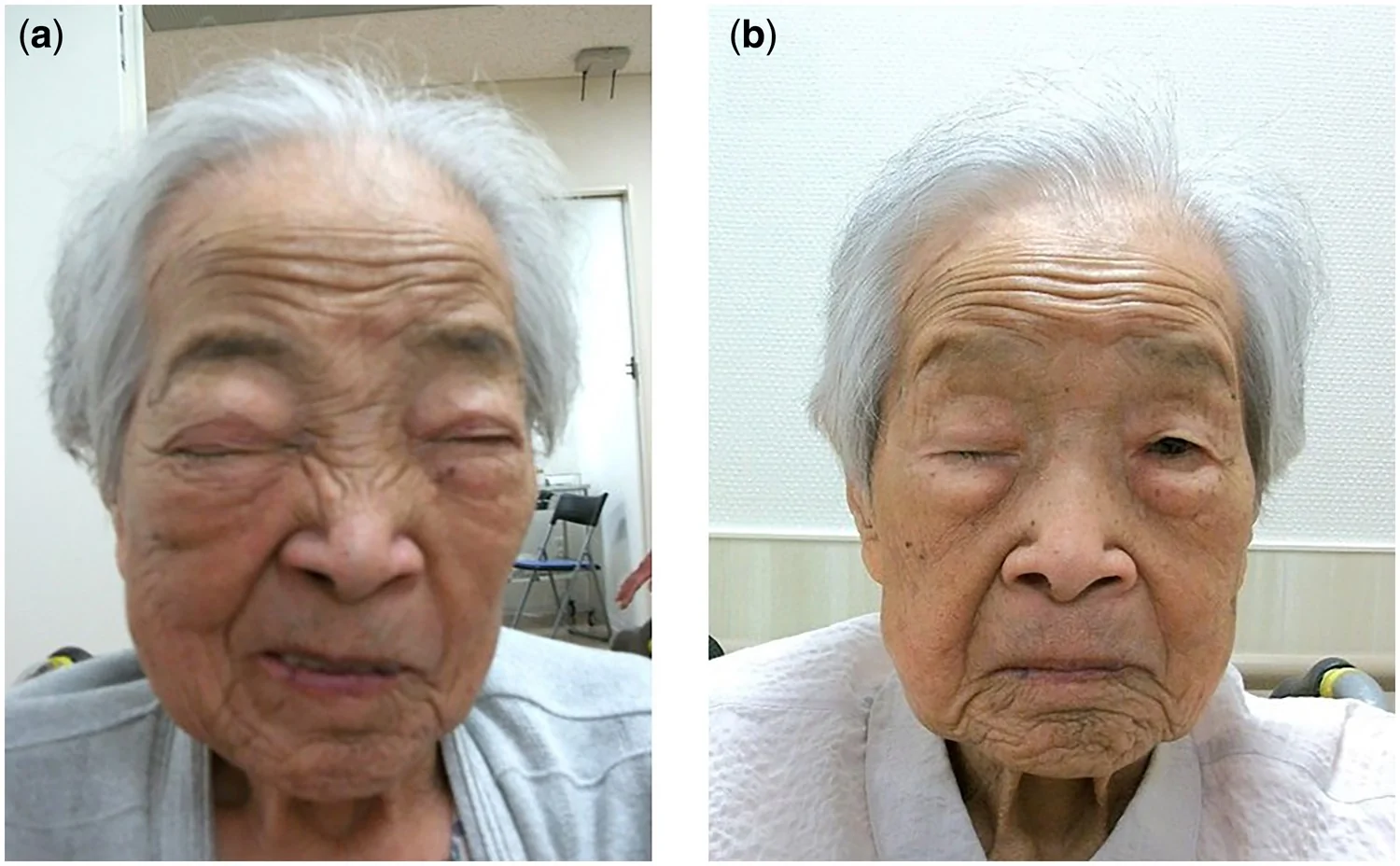
Photos of the face before and 1 year after radiotherapy. (A) A woman in her 90s presented with right exophthalmos, difficulty in opening her eye, and edema. (B) One year after starting treatment, her oculomotor, trigeminal, and facial nerve paralysis has improved.

A woman in her 90s presented to a local clinic with right-sided proptosis, ptosis, diplopia, and nasal obstruction (Figure 1A).

### Image findings

**Figure 2:**
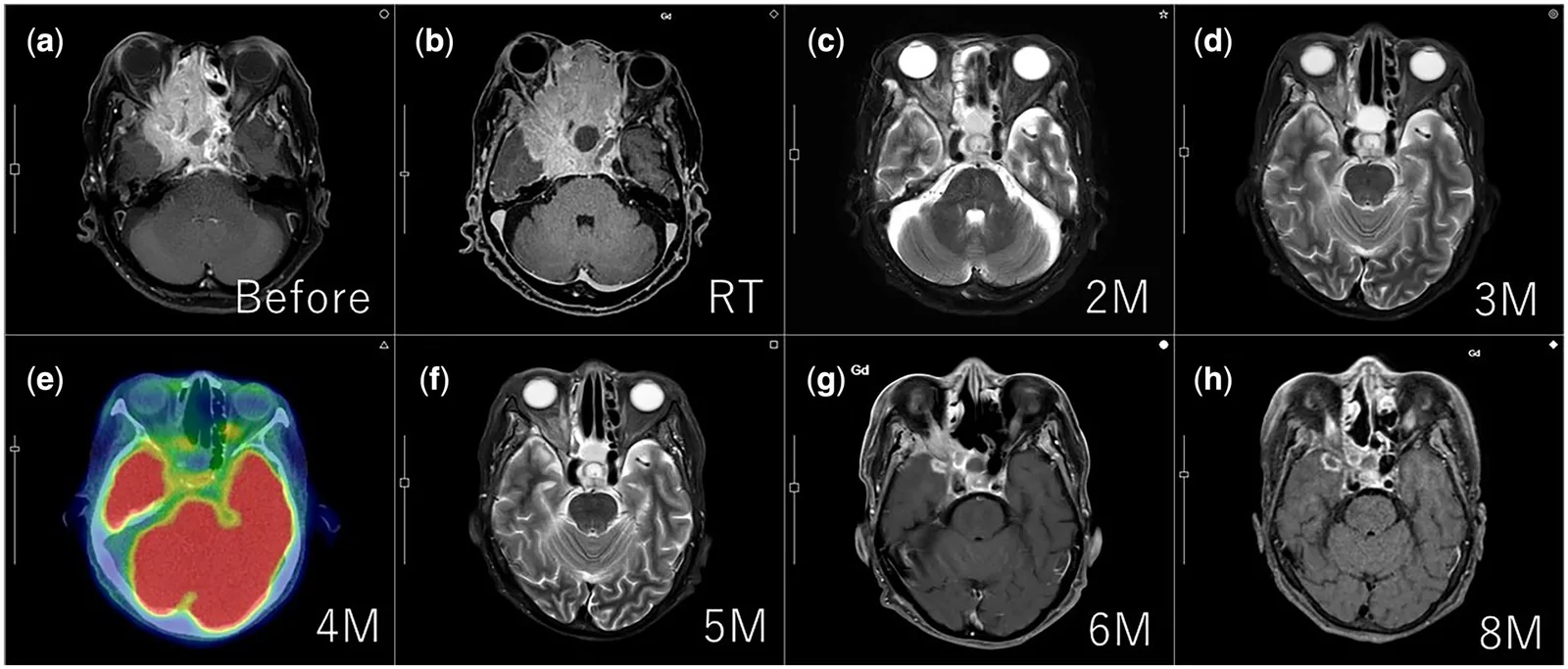
Changes over time. MRI images from A to H (only (E) is fluorodeoxyglucose positron emission tomography). (A) “Before” image at the time of referral to our department. (B) “RT” image at the start of radiation therapy. (C) and onwards: the number of months since treatment is indicated in M (months). (D, E) FDG-PET and (F) No signs of recurrence. (G) Primary lesion remained unchanged and small brain metastases (right temporal lobe) observed. (H) Primary lesion remained unchanged, but brain metastases tended to grow.

Contrast-enhanced computed tomography and magnetic resonance imaging (MRI) revealed a 55 × 75 mm tumor extending from the right ethmoid sinus into the anterior cranial base and orbital cavity (Figure 2). No lymph node metastasis was observed at the time, and the patient was diagnosed with a T4bN0M0 tumor. Biopsy confirmed SCC, and her SCC antigen (SCC-Ag) level was elevated to 53 ng/mL. Her performance status was 3, and she had comorbidities, including heart failure and chronic renal dysfunction, rendering her ineligible for radical surgery or chemoradiotherapy. Prior to treatment, the patient had been taking non-steroidal anti-inflammatory drugs (NSAIDs) and opioid analgesics for pain management.

### Treatment

**Figure 3:**
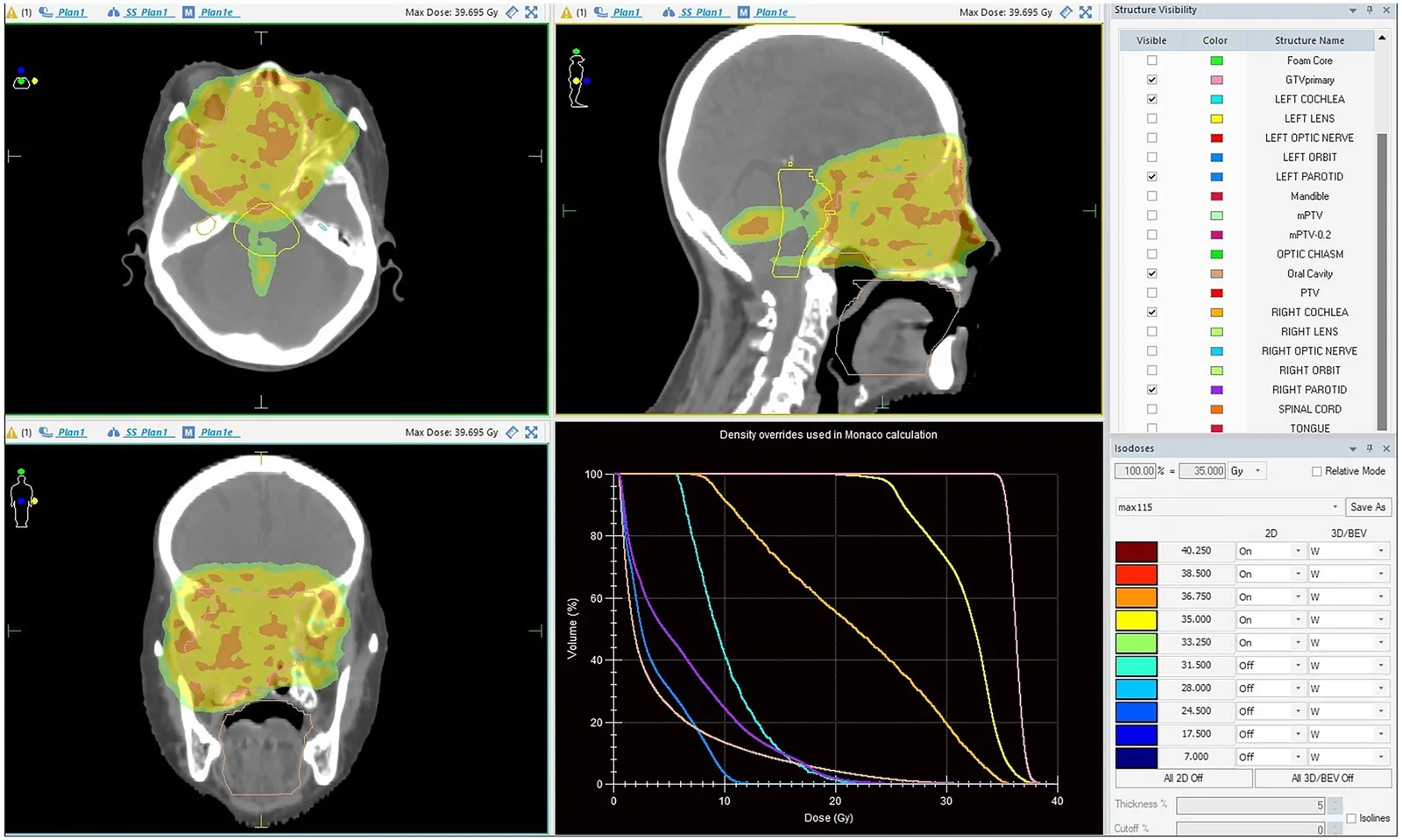
Radiotherapy plan. Yellow: areas covered by 95% of the prescribed dose (35 Gy/5 fr). Orange: areas covered by 100% of the prescribed dose.

For palliative purposes, hypofractionated radiotherapy at 7 Gy × 5 fractions (35 Gy in total) was delivered to the gross tumor volume over 5 consecutive days using intensity-modulated radiotherapy (Figure 3). The 35 Gy in 5 fractions schedule was selected for its favorable biological effective dose (BED10 = 59.5 Gy), its short treatment duration (5 consecutive days) to minimize patient burden, and its established use in palliative head and neck radiotherapy settings. This regimen was preferred over more protracted palliative schedules (eg, 30 Gy in 10 fractions or 20 Gy in 5 fractions) due to the patient’s limited performance status and the need for rapid symptom relief. The plan limited the radiation dose to organs at risk, including the optic nerves, eyes, brainstem, retinae, and temporal lobes. The biological effective dose for the relevant alpha/beta ratio was 42.5 Gy. Although the dose exceeded the tolerance for both lenses, priority was given to tumor control because of the prognosis. The mean doses administered to the right and left lens, parotid gland, and cochlea were as follows: right lens 18.5 Gy, left lens 17.4 Gy, right parotid gland 6.0 Gy, left parotid gland 4.0 Gy, right cochlea 28.2 Gy, and left cochlea 10.2 Gy (all mean doses). The maximum doses to the right optic nerve, left optic nerve, and optic chiasm were 37.1 Gy, 37.1 Gy, and 38.0 Gy, respectively; and the brainstem received a maximum dose of 38.3 Gy. The mean doses to the right and left retinae were 15.2 Gy and 17.0 Gy, respectively.

### Outcomes

Two months post-treatment, proptosis significantly improved. Magnetic resonance imaging revealed an approximately 80% reduction in the tumor volume and partial response. The patient’s vision slightly improved, and analgesic requirements decreased substantially; the patient’s pretreatment regimen of NSAIDs and opioids was reduced to celecoxib alone. MRI follow-up was performed monthly (Figure 2).

At 3 months, a cystic lesion was observed within the irradiated area. Positron emission tomography-computed tomography at 4 months showed no abnormal fluorodeoxyglucose uptake, indicating that the patient showed a complete response (Figure 2E). The SCC level decreased to 0.8 ng/mL. It is important to note that this complete metabolic response reflected local tumor control at the primary site; subsequent disease progression manifested as distant metastases (cervical lymph nodes and brain), representing systemic rather than local recurrence.

At 6 months, the SCC level slightly increased to 1.6 ng/mL. However, imaging showed no recurrence. At 8 months, the SCC level increased to 3.6 ng/mL. MRI revealed bilateral cervical lymph node metastases and a 6-mm brain metastasis in the right temporal lobe (Figure 2G). However, the patient remained asymptomatic. Mild osteonecrosis of the right maxilla was observed and followed conservatively.

At 12 months, mild progression of the metastases was observed without neurological symptoms. Stereotactic radiosurgery (20 Gy in 1 fraction) was delivered to the metastatic lesions in the brain (Figure 2H). The patient declined further cervical node irradiation and opted for palliative care under the guidance of her family doctor. The prolonged period of local disease control achieved by the initial hypofractionated radiotherapy, which preserved the patient’s performance status over 12 months, was likely instrumental in enabling the subsequent stereotactic radiosurgery for brain metastases and thereby contributing to overall disease stabilization.

Only grade 1 dermatitis and mild fatigue were observed as acute toxicities during the 12-month follow-up period. A limitation of this report is that the 12-month observation period precludes comprehensive assessment of late radiation toxicities, such as radiation-induced optic neuropathy, cataract progression, or further development of osteoradionecrosis, all of which may manifest beyond this timeframe. The patient’s initial QOL and symptoms (pain and appearance) improved markedly. At the end of follow-up, no evidence of local recurrence was detected on MRI or FDG-PET, and the patient continued follow-up care with her family doctor.

## Discussion

As described in the “Introduction,” ethmoid sinus carcinoma is often diagnosed at an advanced stage because of its anatomical location and nonspecific early symptoms. Surgical resection combined with postoperative radiotherapy remains the standard treatment approach for locoregional control; however, complete (R0) resection is often difficult because of the proximity of the tumor to critical structures including the orbit and skull base, leading to a high rate of residual disease and local failure.^1–4^

In our case, radical surgery and chemoradiotherapy were unfeasible considering the patient’s advanced age and poor general condition. Therefore, hypofractionated palliative radiotherapy was selected, which provided satisfactory symptom relief and tumor shrinkage. This is consistent with the findings of Aljabab et al^4^, who described the efficacy of split-course high-dose radiotherapy in similar scenarios.

Briefly, other intensified approaches such as selective intra-arterial cisplatin infusion with concurrent radiotherapy (RADPLAT) and particle therapy (proton or carbon-ion) have demonstrated promising local control for sinonasal malignancies in patients suitable for more aggressive treatment.^5^ However, a detailed discussion of these modalities is beyond the scope of this palliative case report, as they are not applicable in patients such as ours who are medically unsuitable for radical treatment.

Patel et al^7^ demonstrated in a meta-analysis that particle therapy (proton and carbon-ion) offered improved 5-year local control and overall survival with reduced toxicities compared with photon therapy for paranasal sinus malignancies.^6^ Given the anatomical complexity of the ethmoid sinus, particle therapy may be particularly beneficial in cases where surgery is unfeasible or when residual disease persists postoperatively.

Kacorzyk et al^3^ also highlighted the importance of aggressive salvage strategies for sinonasal non-SCC, where surgery following primary treatment failure could restore long-term survival in selected patients. Similarly, Wang et al^2^ observed that combining surgery with PORT could result in satisfactory long-term outcomes despite the high rates of R1 or R2 resections in frontal sinus malignancies; however, local recurrence remained the predominant failure pattern.

In our case, hypofractionated photon radiotherapy alone achieved meaningful palliation with minimal toxicity. Importantly, the durable local control enabled subsequent stereotactic radiosurgery for brain metastases at 12 months, which may have contributed to the overall prolongation of disease stabilization. These findings support the role of localized, short-course radiotherapy as a pragmatic option in patients who are medically or anatomically unsuitable for radical interventions. Moving forward, integration of particle therapy, radiation therapy and concomitant intra-arterial cisplatin infusion (RADPLAT) or combined modality strategies may further refine treatment approaches for this difficult-to-treat disease.

## Conclusion

In the present case of advanced, unresectable ethmoid sinus SCC, hypofractionated radiotherapy targeting only the gross tumor volume (7 Gy × 5 fractions; 35 Gy in total) resulted in effective symptom relief and radiological tumor reduction. Although the intent of this treatment was not curative, it successfully improved the patient’s QOL with minimal toxicity.

This case supports the utility of localized, short-course radiotherapy as a practical and effective palliative option in patients regarded unsuitable for radical interventions. Moving forward, further clinical experience and comparative studies, especially those involving particle therapy, are needed to refine treatment strategies for this challenging and rare disease.

## Learning points

- Ethmoid sinus squamous cell carcinoma is rare, and treatment in very elderly patients poses significant challenges.
- Hypofractionated radiotherapy (35 Gy/5 fractions) can provide rapid and durable palliation of symptoms such as obstruction and bleeding.
- Careful selection of patients and individualized treatment planning are essential to achieve palliation with minimal toxicity.
- Palliative radiotherapy remains an important option for frail and elderly patients where curative treatment is not possible.

## Data Availability

The data supporting the findings of this case report are included within the article. Further inquiries can be directed to the corresponding author.
